# Primary Amenorrhea With Bilateral Endometriomas in a Teenager Girl With Cervical Hypoplasia and Vaginal Agenesis

**DOI:** 10.1002/ccr3.72049

**Published:** 2026-02-19

**Authors:** Shahzeen Irshad, Maryam Irshad, Rida Fatima Gilani, Mahnoor Fatima, Abdul Sattar Anjum, Sehrish Irshad

**Affiliations:** ^1^ Nishtar Medical University Multan Pakistan; ^2^ Shaheed Ziaur Rahman Medical College Bogura Bangladesh

**Keywords:** amenorrhea, cervicovaginal agenesis, Mullerian duct anomalies, pelvic pain

## Abstract

A rare case of primary amenorrhea caused by cervical hypoplasia and vaginal agenesis highlights the critical need for early diagnosis and timely surgical intervention to prevent severe complications such as hematometra, endometriosis, infections, infertility, and a staged multidisciplinary approach optimizes outcomes in these rare, complex presentations.

## 1 Case Image

A 16‐year‐old girl presented to the gynecological emergency with primary amenorrhea and progressively worsening cyclical lower abdominal pain. The pain began at age 11 as intermittent cramps, later on evolved into a constant, severe ache radiating across the pelvis with transient relief from oral and intravenous analgesics and no other associated symptoms.

On physical examination, she appeared healthy with normal height (5′3″) and build and exhibited fully developed secondary sexual characteristics. Abdominal palpation revealed no significant findings. Examination of external genitalia confirmed normally formed labia majora, labia minora, and clitoris but revealed only a 2–3 cm blind‐ending vaginal pouch.

**FIGURE 1:**
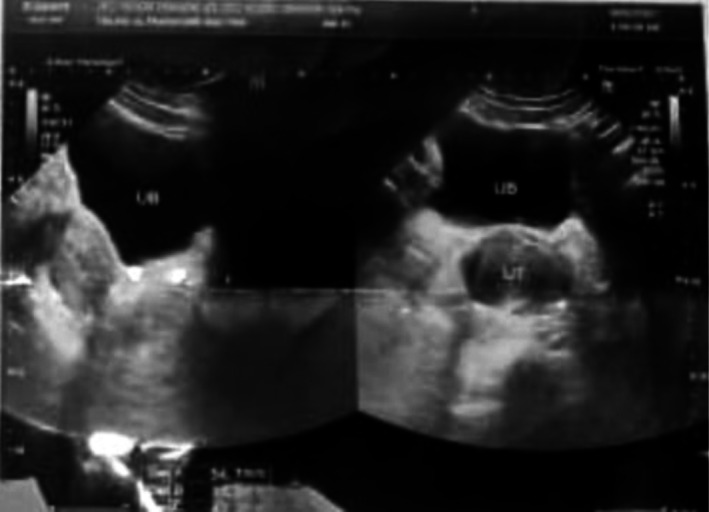
Transabdominal pelvic ultrasound showing hematometra in a 16‐year‐old female with cervicovaginal agenesis. The sagittal and transverse views demonstrate a distended uterine cavity (UT) filled with homogeneous hypoechoic fluid, consistent with hematometra. The urinary bladder (UB) appears anterior to the uterus. The cervical canal and vaginal canal are not visualized, supporting the diagnosis of cervicovaginal agenesis as the underlying cause of obstructed menstrual outflow.

**FIGURE 2:**
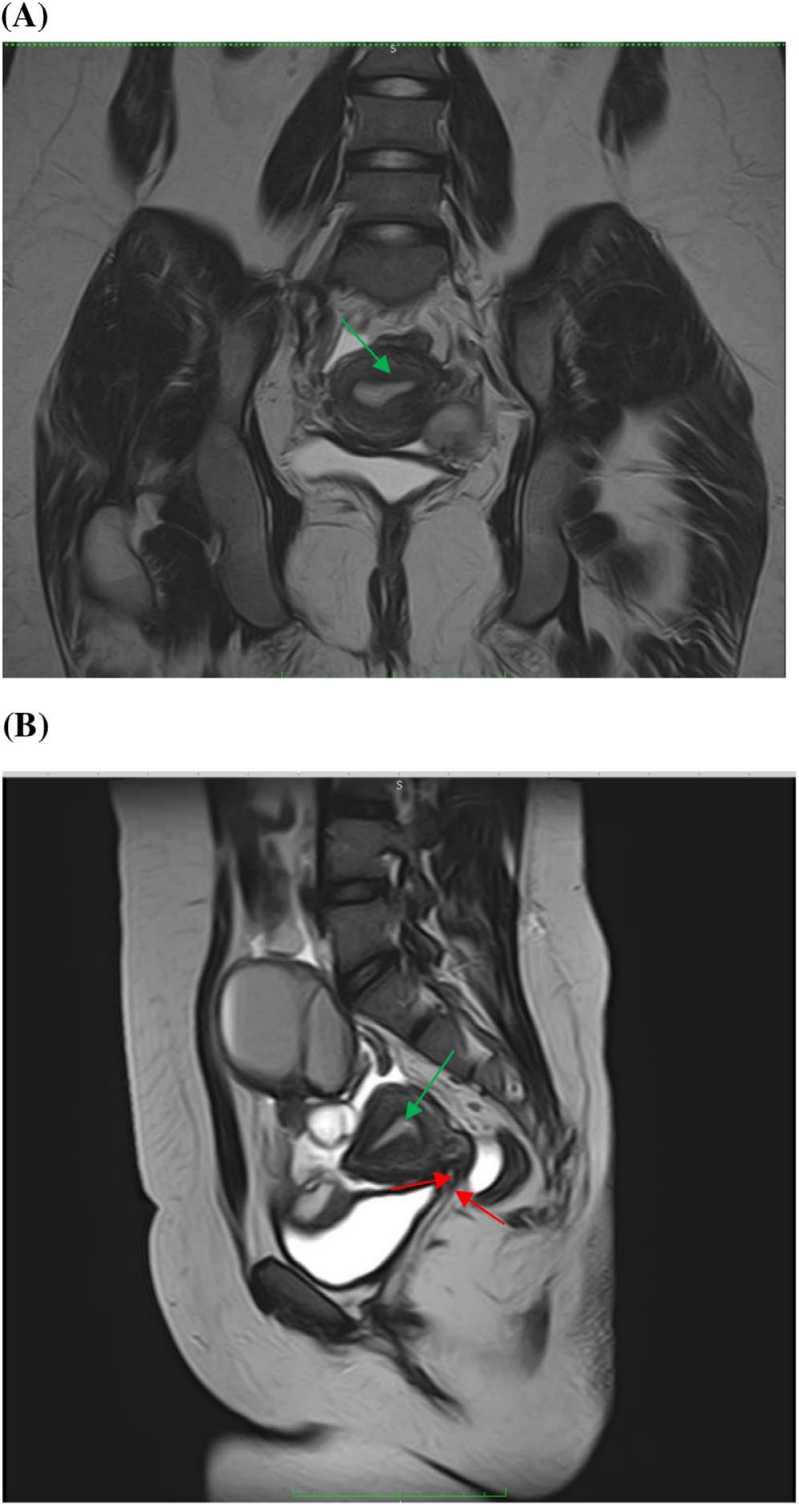
(A) Coronal T2 (B) Sagittal T2. MR imaging findings showing cervical hypoplasia and vaginal agenesis (red arrows) and expanded uterine cavity (green arrows).

Transabdominal ultrasound showed a distended uterine cavity filled with echogenic fluid (Figure 1), and pelvic MRI demonstrated an anteverted uterus (5.5 × 4.3 cm) containing multicystic fluid collections of high T1 and T2 signal intensity compressing the bladder and uterine fundus with bilateral endometriotic cysts (Figure 2). These findings supported a diagnosis of cervical hypoplasia with total or partial vaginal agenesis (AFS Class 1/U5aC4V4) [1] complicated by cryptomenorrhea and endometriosis.

After multidisciplinary discussion, total abdominal hysterectomy was recommended as definitive management but the patient and her mother declined due to concerns regarding future fertility.

Because conservative measures had failed, laparotomy was planned. Intraoperatively, both ovaries were distorted by endometriotic cysts measuring 8 × 6 cm on the right and 6 × 7 cm on the left; the fallopian tubes were densely adherent to these masses and surrounding pelvic structures. The cysts were removed, and the remaining ovarian cortex was meticulously reconstructed to preserve ovarian function.

Her post‐operative recovery was uneventful; she was discharged on continuous combined oral contraceptives for 6 months to suppress menstruation and reduce cyst recurrence. Definitive cervicovaginal reconstruction will be an option when the family is ready to revisit surgical options.

Cervical agenesis or hypoplasia is an extremely rare disorder with a prevalence of 1:80,000 to 1:100,000 and about half of these cases coexist with vaginal aplasia [2]. It is a type of Müllerian duct anomalies that arise from disrupted development, fusion, or septal resorption of the paramesonephric ducts and urogenital sinus, producing a spectrum of defects, from asymptomatic variants to severe agenesis such as Mayer–Rokitansky–Küster–Hauser (MRKH) syndrome or obstructive lesions that demand urgent correction [1].

Three‐dimensional ultrasound and magnetic resonance imaging (MRI) remain the preferred modalities for diagnosis of mullerian anomalies [2]. The clinical presentation and treatment for Müllerian malformations are directly related to the anatomy of the defect. Malformations that obstruct the menstrual flow should be treated rapidly by doing a suitable surgical procedure for each case. Patients with cervical hypoplasia and vaginal agenesis may undergo uterovaginal anastomosis, vaginoplasty, or vaginal dilatation through diverse techniques [1]. Overall, the diagnosis and treatment of such conditions present as a challenge both for patients and health care professionals.

This study underscores the need for deeper genetic and embryologic insights into rare Müllerian anomalies, and establishing standardized guidelines for early diagnosis and management of atypical cases.

## Author Contributions

**Shahzeen Irshad:** conceptualization, methodology, supervision, writing – original draft, writing – review and editing. **Maryam Irshad:** conceptualization, data curation, investigation, writing – original draft. **Rida Fatima Gilani:** conceptualization, data curation, investigation, writing – original draft. **Mahnoor Fatima:** conceptualization, data curation, investigation, writing – original draft. **Abdul Sattar Anjum:** conceptualization, project administration, writing – original draft. **Sehrish Irshad:** conceptualization, investigation, writing – review and editing.

## Consent

Written informed consent for the publication of the patient's clinical information and imaging was obtained from the patient as well as the patient's guardian.

## Conflicts of Interest

The authors declare no conflicts of interest.

## Data Availability

The data that supports the findings of this study are available in the Supporting Information of this article.
